# Quantitative Imaging of Regional Aerosol Deposition, Lung Ventilation and Morphology by Synchrotron Radiation CT

**DOI:** 10.1038/s41598-018-20986-x

**Published:** 2018-02-23

**Authors:** L. Porra, L. Dégrugilliers, L. Broche, G. Albu, S. Strengell, H. Suhonen, G. H. Fodor, F. Peták, P. Suortti, W. Habre, A. R. A. Sovijärvi, S. Bayat

**Affiliations:** 1 Department of Physics, University of Helsinki, Helsinki, Finland; 2 Helsinki University Central Hospital Medical Imaging Center, Helsinki, Finland; 3 Department of Pediatric Intensive Care, Amiens University Hospital, Amiens, France; 4 Hedenstierna Laboratory, Department of Surgical Sciences, Uppsala University, Uppsala, Sweden; 5 Anesthesiological Investigations Unit, University Hospitals of Geneva, Geneva, Switzerland; 6 Department of Medical Physics and Informatics, University of Szeged, Szeged, Hungary; 7 Department of Clinical Physiology and Nuclear Medicine, Helsinki University Central Hospital and University of Helsinki, Helsinki, Finland; 8 University of Grenoble EA-7442 RSRM Laboratory and Department of Clinical Physiology, Sleep and Exercise, Grenoble University Hospital, Grenoble, France

## Abstract

To understand the determinants of inhaled aerosol particle distribution and targeting in the lung, knowledge of regional deposition, lung morphology and regional ventilation, is crucial. No single imaging modality allows the acquisition of all such data together. Here we assessed the feasibility of dual-energy synchrotron radiation imaging to this end in anesthetized rabbits; both in normal lung (n = 6) and following methacholine (MCH)-induced bronchoconstriction (n = 6), a model of asthma. We used K-edge subtraction CT (KES) imaging to quantitatively map the regional deposition of iodine-containing aerosol particles. Morphological and regional ventilation images were obtained, followed by quantitative regional iodine deposition maps, after 5 and 10 minutes of aerosol administration. Iodine deposition was markedly inhomogeneous both in normal lung and after induced bronchoconstrition. Deposition was significantly reduced in the MCH group at both time points, with a strong dependency on inspiratory flow in both conditions (R^2^ = 0.71; p < 0.0001). We demonstrate for the first time, the feasibility of KES CT for quantitative imaging of lung deposition of aerosol particles, regional ventilation and morphology. Since these are among the main factors determining lung aerosol deposition, we expect this imaging approach to bring new contributions to the understanding of lung aerosol delivery, targeting, and ultimately biological efficacy.

## Introduction

The lung presents a large surface area in contact with the ambient atmosphere, and is the first barrier against environmentally inhaled particles, including atmospheric particulate matter and aerosols. Organic aerosols can carry antigenic compounds such as pollens, microorganisms and microbial toxins as well as environmental pollutants^1,2^. On the other hand, inhaled therapy is the mainstay of treatment in obstructive lung diseases such as asthma, COPD and cystic fibrosis^3^. This route of treatment has the advantage of minimizing systemic versus pulmonary effects, while having a rapid onset of action^4^. Moreover, aerosol inhalation is increasingly being assessed as a route to administer other therapeutic agents such as viral vectors for gene therapy; peptides for the treatment of lung diseases such as vasoactive intestinal peptide to treat pulmonary hypertension; glutathione to treat cystic fibrosis; granulocyte-macrophage colony-stimulating factor to treat pulmonary alveolar proteinosis; calcitonin for postmenopausal osteoporosis; or insulin to treat diabetes^5^.

Aerosol particle deposition in the lower respiratory tract depends on a number of factors, including mass median aerodynamic diameter (MMAD), the inspiratory flow velocity and the flow regime, and the lung airway morphology^4^. In the first few generations of the bronchial tree where the airflow velocity is relatively higher, aerosol particle deposition occurs mainly through inertial impaction, which is due to the inability of particles to follow rapid changes in gas flow direction^6^. In this regard, airway narrowing due to bronchoconstriction, increased mucus secretion or remodeling as seen in obstructive respiratory diseases is a key determinant of the pattern of aerosol deposition in pathologic conditions, since the local gas flow velocity and likelihood of turbulent flow both increase in narrowed airways. Airway narrowing, can be very unevenly distributed among airway generations and can thereby contribute to the heterogeneity of aerosol deposition^7^. This may in turn cause substantial differences in the regional delivery of medications to the lung. Currently, different strategies are being developed in order to target aerosol particles to specific regions of the lung, preferentially those that are more affected by a disease process. The success of such strategies in translating into clinical applications critically depends on imaging techniques allowing for both the quantitative determination of aerosol deposition, and the morphology of the bronchial tree, ideally using a single imaging modality with a high spatial resolution in order to also be applicable in preclinical small animal models. Traditional methods have mainly used radionuclide imaging of radioactively labeled aerosols, such as Single Photon Emission Computed Tomography (SPECT) or Positron Emission Tomography (PET) which are limited by their spatial resolution^4^. Also, because these techniques do not allow imaging lung morphology, they are combined with computed x-ray tomography (CT), which requires co-registration of the acquired images on the combined instruments.

Here, we describe a novel application of synchrotron radiation K-edge subtraction CT imaging, for the quantitative mapping of lung aerosol particle deposition; lung morphology and regional lung ventilation. The latter is important for understanding aerosol deposition and its biological effects particularly in conditions of spatially inhomogeneous lung function, as is the case in lung diseases. Acquiring these data together has the potential of bringing new insight into the distribution of aerosol particle deposition. The imaging technique uses a single quantitative modality, with a comparatively high spatial as well as temporal resolution, allowing for repeated image acquisitions in *in vivo* preclinical models. We present data demonstrating the feasibility of the technique for quantitatively mapping aerosol deposition in anesthetized rabbits with normal lungs or following acute bronchoconstriction, a model of asthma. Our data demonstrate that quantitative imaging of the deposition of aerosol particles in the lung together with regional ventilation and morphology is feasible with KES CT imaging.

## Results

**Figure 1:**
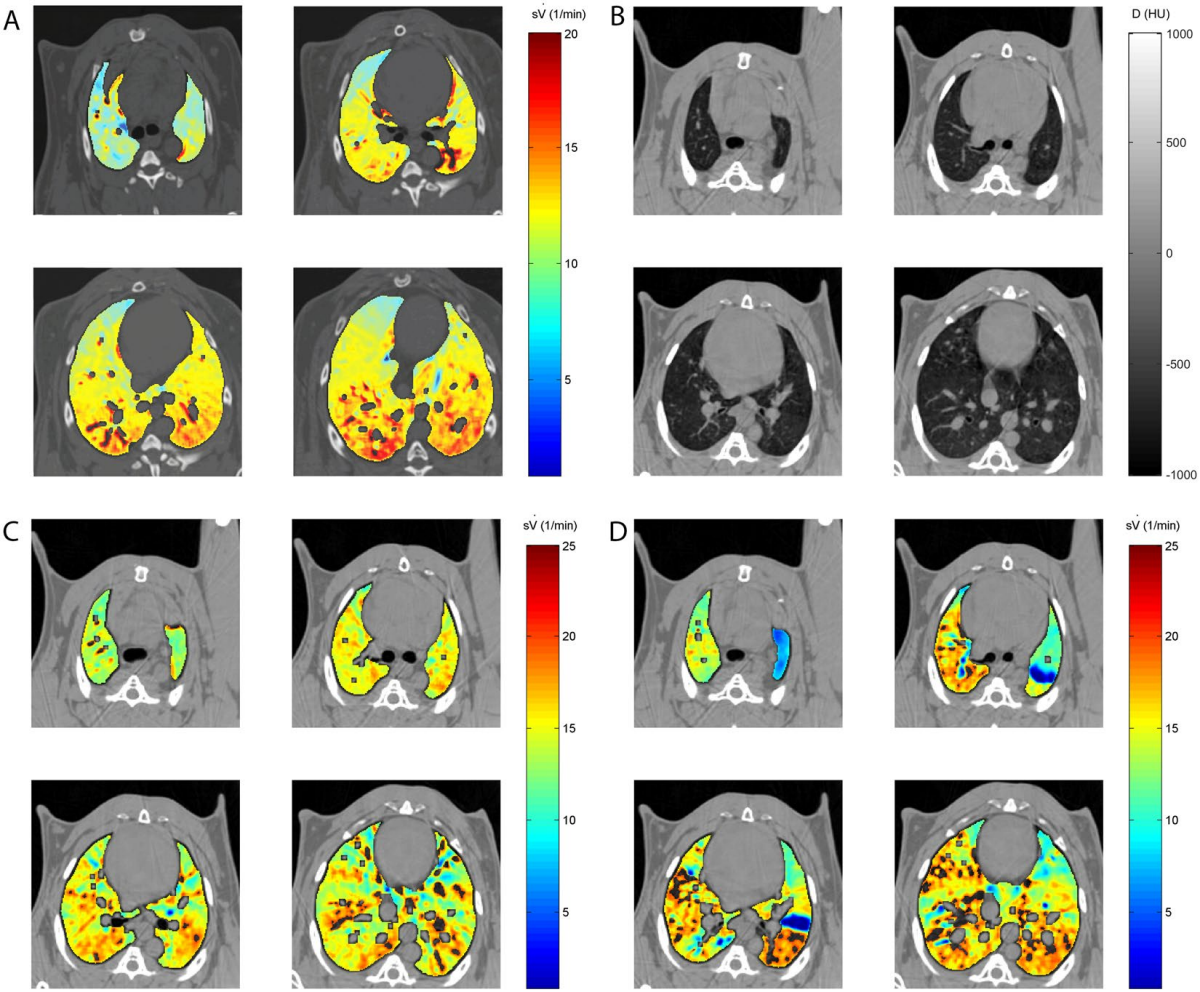
KES CT images of specific lung ventilation (${\rm{s}}\dot{V}$), in one representative control rabbit (**A**); lung morphology (**B**) and ${\rm{s}}\dot{V}$ images in a MCH-challenged animal at baseline (**C**) and during the challenge (**D**). Note the constriction of central conducting airways and small peripheral ventilation inhomogeneities during MCH infusion.

**Figure 2:**
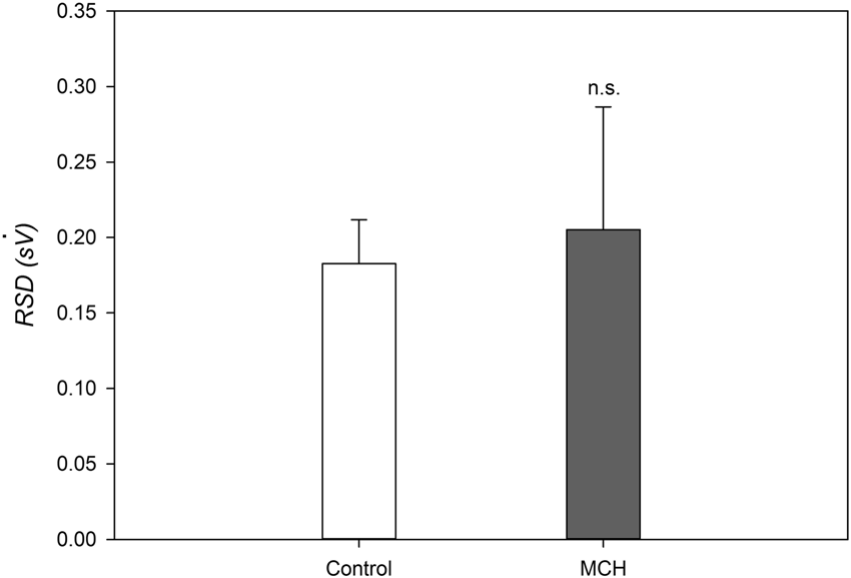
Ventilation inhomogeneity based on the RSD of ${\rm{s}}\dot{V}$. Data are m ± SD, n = 6 in each group.

**Figure 3:**
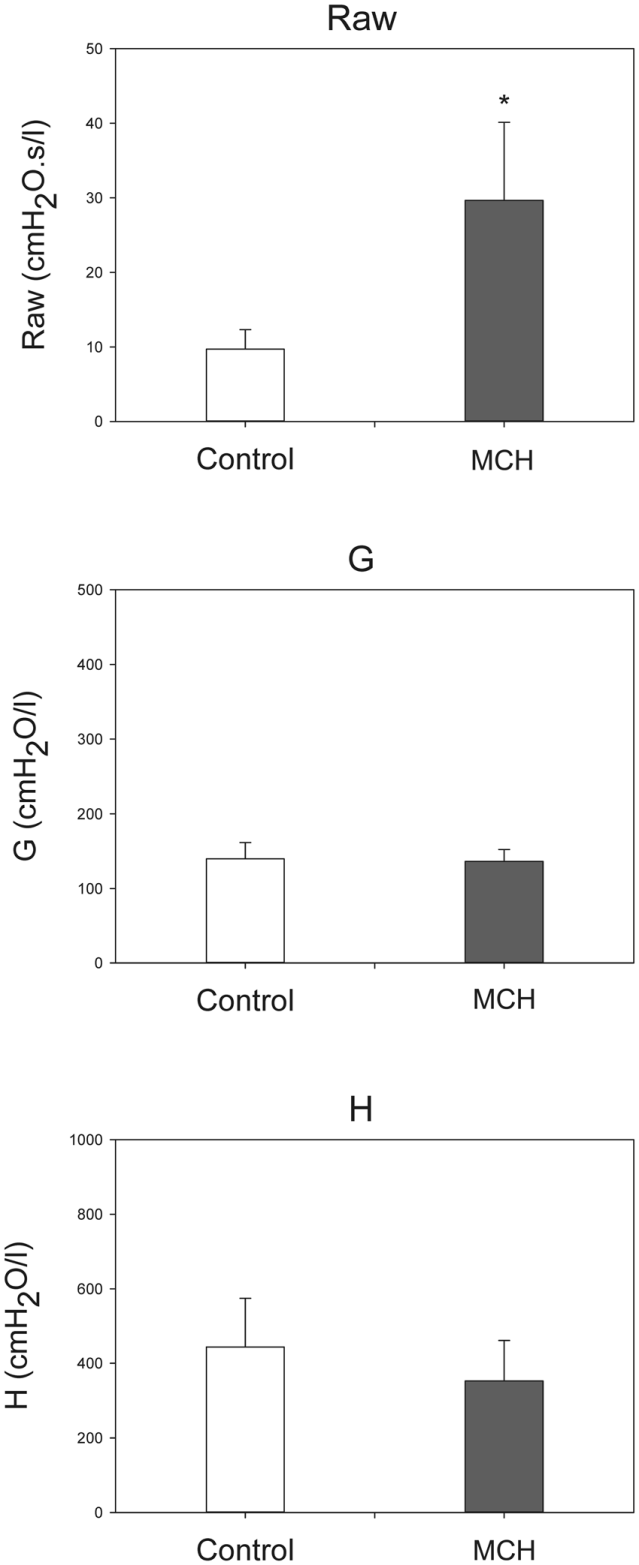
Respiratory mechanical parameters measured by forced oscillations. Data are m ± SD, n = 6 in each group. *p < 0.05 vs. control.

**Figure 4:**
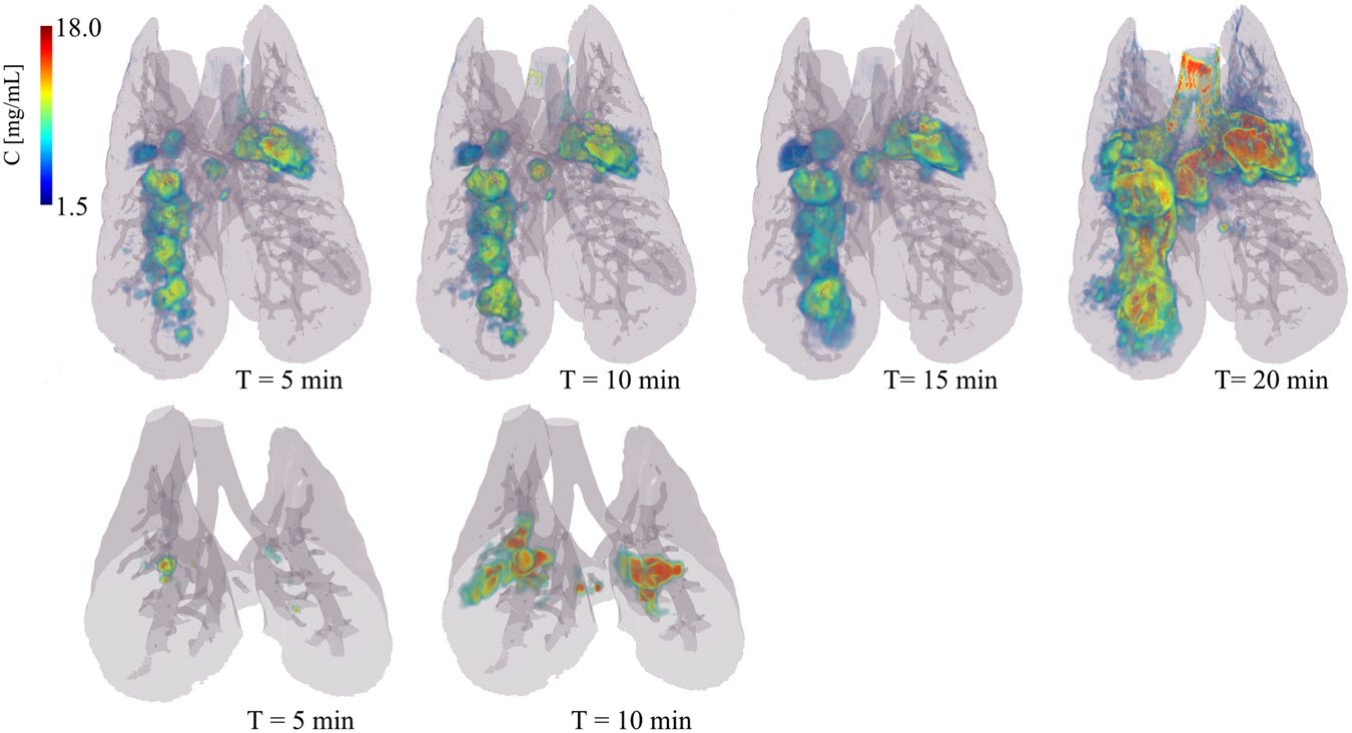
Three-dimensional rendering of the regional deposition of iodine expressed as concentration (mg/ml) versus the duration of inhalation in a representative control (top row, whole lung) and an MCH animal (bottom row, 35 mm vertical height). Grey colour: surface rendering of lung and conducting airway morphology; dark grey shows the projection of segmented conducting airways. Values below 1.5 mg/ml, were not represented in order to better reveal focal areas of strong iodine deposition. Note the strongly inhomogeneous pattern and increasing amount of deposition with time in the control animal, and a similarly inhomogeneous pattern but less overall deposition in the MCH animal.

**Figure 5:**
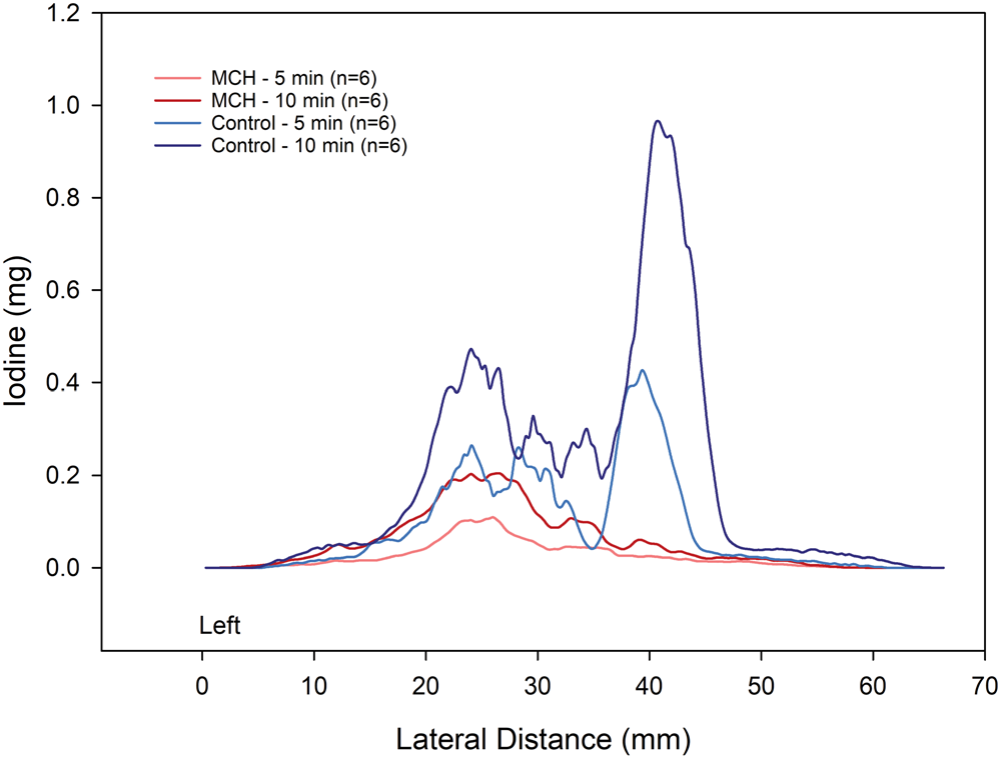
Comparison of regional iodine deposition in sample control and MCH – challenged animals, projected along the left-right axis, at 5 and 10 min of aerosol inhalation. Note the regional and inter-subject inhomogeneity of deposition, and the spatial similarity of deposition pattern between 5 and 10 min time points.

**Figure 6:**
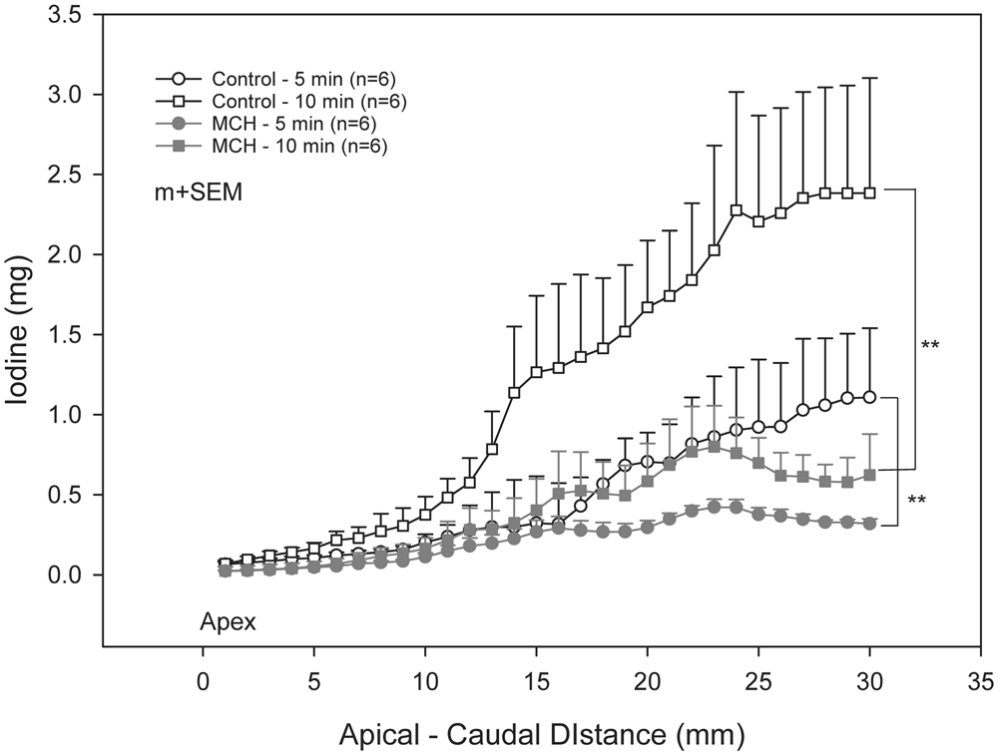
Comparison of regional iodine deposition in control and MCH – challenged animals represented as mean ± SEM total iodine per image level vs. apical – caudal distance, after 5 and 10 min of aerosol inhalation. **p < 0.001.

**Figure 7:**
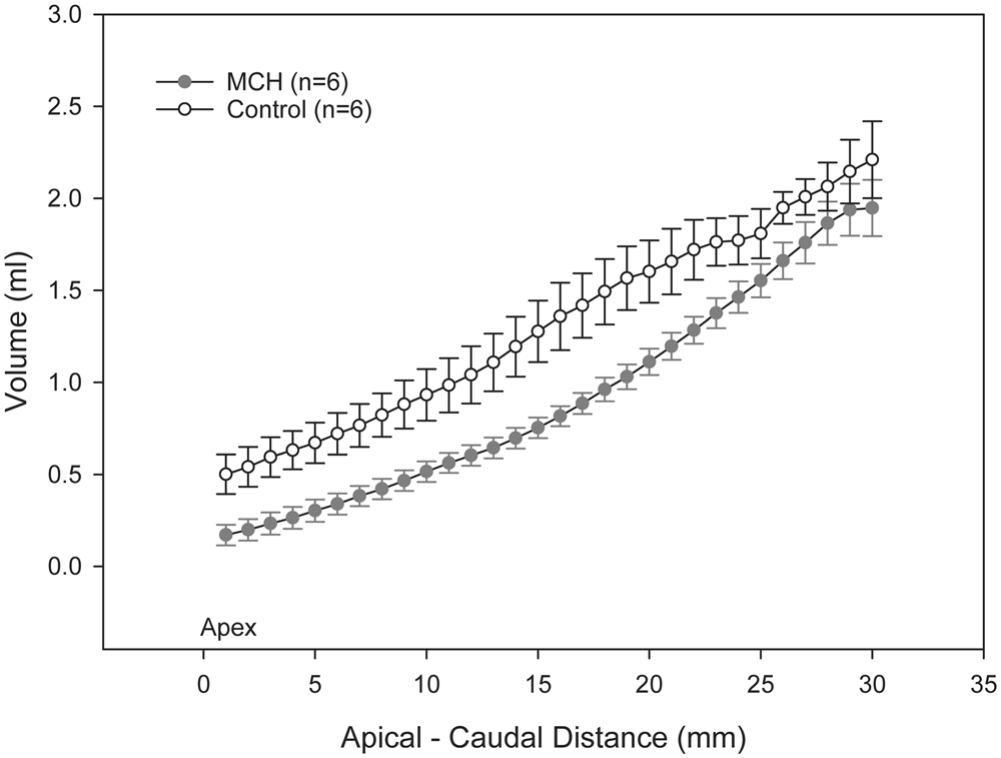
Comparison of regional lung volume in control and MCH – challenged animals represented as mean ± SEM per image level vs. apical – caudal distance. *p < 0.05.

**Figure 8:**
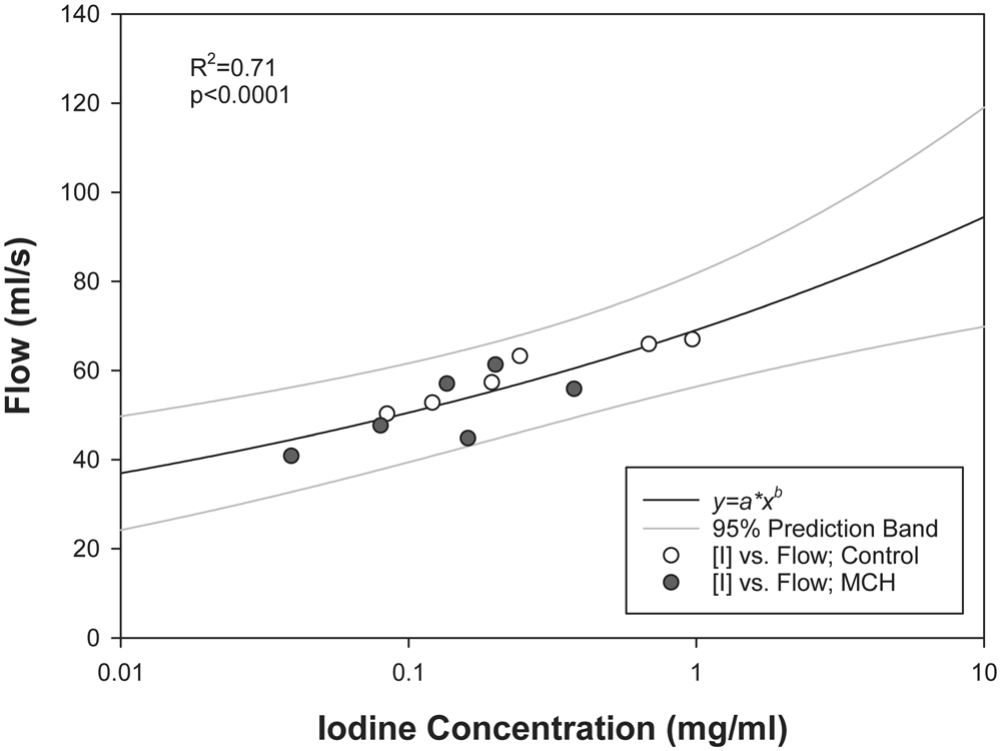
Relation between mean inspiratory air flow during inhalation and mean iodine concentration within the imaged lung. *Black line*: Fitted power function; *Grey lines*: prediction limits.

Composite images showing maps of specific lung ventilation in 4 axial image levels in a representative control and a methacholine (MCH)-challenged animal are shown in Fig. 1. The MCH challenges caused little inhomogeneity in peripheral lung ventilation, in line with our previous findings in this experimental model^8–10^. Indeed, the residual standard deviation of specific ventilation (s), or ventilation per regional gas volume, which indicates how fast a given lung region fills with inhaled gas, was only slightly and non-significantly increased (Fig. 2). The differences in respiratory mechanical parameters induced by MCH infusion are summarized in Fig. 3. Methacholine infusion increased airway resistance (Raw), without inducing significant changes in lung tissue damping (G) or elastance (H). Damping reflects oscillatory energy dissipation within the respiratory tissues^11^. This parameter increases with lung tissue resistance and may be increased by mechanical heterogeneity of the parenchyma, while H reflects elastic energy storage within the respiratory tissues reflecting respiratory tissue stiffness^12^ or loss of volume e.g. through airway closure^8^. Figure 4 shows a composite 3D-rendering of the lung structure as well as the regional iodine concentration within the imaged lung. The contours of the first few generations of conducting airways as well as the outer surfaces of the lung can be visualized. The inhomogeneous deposition of the aerosol can be observed. Also, increasing deposition with the duration of aerosol inhalation could be evidenced through repeated KES imaging. Interestingly, the iodine-containing aerosol particles seemed to deposit on focal points or “hot-spots” whose concentration and extent increased with repeated aerosol administration. This point is further illustrated by the amount of iodine deposition projected along the lateral axis in representative animals of each group, shown in Fig. 5. The total amount of iodine deposition at each axial slice along the apical-caudal distance was computed in both groups and time intervals, and is shown in Fig. 6. Deposition increased with administration time in both groups, but was significantly lower in the MCH-challenged group at both time intervals. The increasing regional deposition in the apical-caudal direction mirrored that of lung volume, as shown in Fig. 7. We did not find a significant relation between regional iodine concentration or mass deposition and specific ventilation. The overall lung volume was slightly but significantly decreased in the MCH-challenged group. Regression analysis did show however, a strong relation between inspiratory air flow and iodine concentration in the imaged lung (Fig. 8). This relation was best depicted by a power function. Aerosol particle size measurements showed a median aerodynamic diameter of 2.97 µm with a geometric standard deviation of 0.17.

## Discussion

The goal of this study was to test the feasibility of synchrotron KES CT imaging, for visualizing and quantifying the regional lung deposition of aerosol particles. Our data in this preclinical model in anesthetized and ventilated rabbit, demonstrate for the first time that KES imaging not only allows measuring aerosol deposition distribution, but also lung morphology and regional lung ventilation data can be obtained in the same animal with the same modality. The importance of imaging the regional distribution of inhaled aerosol is underscored by the fact that regional rather than the total lung deposition determines the clinical efficacy of inhaled treatments^13,14^. On the other hand, the morphology of the branching airways and alveoli is an important determinant of aerosol particle deposition^4^. Unlike techniques using radionuclides such as Scintigraphy, SPECT or PET, or which typically need to be combined with CT imaging to elucidate the lung morphology, here the lung structure can be directly imaged within a single dataset that simultaneously yields the contrast element distribution. This precludes the necessity for image registration between the different imaging modalities which is a significant advantage. Moreover, our study shows that aerosol deposition distribution can be imaged repeatedly to obtain data on the kinetics of regional deposition with time, or to determine how interventions can change the deposition pattern of aerosol particles. Using this technique, various preclinical models of lung diseases can be studied such as induced bronchoconstriction which mimicked an acute asthma attack. Here, we used aerosolized iomeprol which is a routinely employed intravenous contrast agent to image the deposition of aerosol particles. However, with the current design and capabilities of the x-ray optical setup of the ESRF-ID17 beamline, different elements with K-absorption edges ranging approximately between 20 and 100 KeV such as: silver, indium, barium, gadolinium, holmium, or gold, among other elements, can be used as tracers. Modifications in X-ray monochromator design may allow acquiring images with a wider energy range of the incident beam spectrum, comprising the K-edges of elements such as xenon and iodine simultaneously. This would allow imaging these elemental distributions Together. Alternatively, using an energy-resolving X-ray detector would serve the same purpose, allowing for multi-energy or spectral imaging^15^.

Previously, computed tomography with a standard x-ray source has been used to study iodine aerosol deposition in a preclinical rabbit model, but as an indicator of regional ventilation distribution^16^. Due to beam hardening of polychromatic x-rays however, standard CT techniques are limited in their ability to quantify aerosol deposition. Alternatively, fluorescent imaging has been used to study the deposition of aerosol particles in lung of mice^17^. Although fluorescence imaging has excellent contrast sensitivity, it is non-quantitative, it is limited by the depth of tissue structures that can be imaged and by phenomena such as tissue absorption, scattering and autofluorescence^18^. Moreover, it does not allow imaging the lung morphology.

Regional ventilation distribution is an important determinant of the peripheral deposition of aerosol particles in the range of 1 to 8 µm^14^. Our data show that ventilation distribution can be imaged and measured quantitatively using the wash in of stable Xe gas, almost simultaneously to aerosol deposition since switching from iodine to Xe K-edge was performed in approximately 2 minutes^19^. This time interval depends on the x-ray optical setup; a wider energy gap between the 2 radiation beams produced with an alternative optical setup would theoretically allow simultaneous quantification of both ventilation and aerosol deposition.

**Figure 9:**
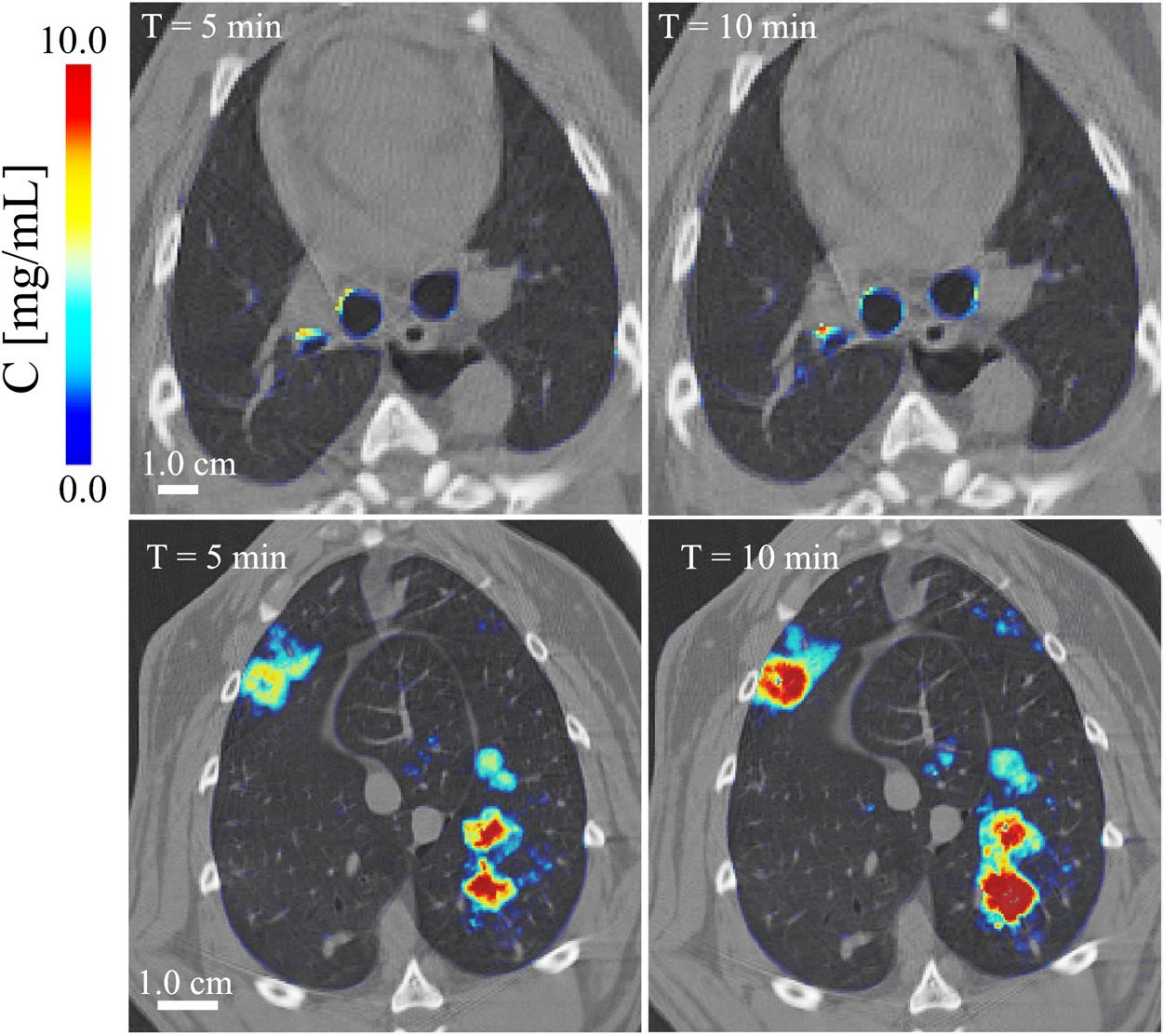
Composite cross-sectional images showing lung morphology and iodine deposition in central conducting airways (*top*) and in the lung periphery (*bottom*) after 5 and 10 min of aerosol inhalation, in one control animal.

In this study, the heterogeneity of regional ventilation distribution did not increase significantly following MCH infusion, in line with our previous findings^8,20,21^. The observed heterogeneous pattern of aerosol deposition, the lack of a significant correlation with regional specific ventilation, and the strong correlation of iodine deposition with the mean inspiratory airflow suggest that inertial impaction may have been the main mechanism of deposition. This mechanism is due to the inability of coarse aerosol particles to follow rapid directional changes in the air stream, mainly at airway bifurcations^14^ and is determined by the air flow velocity. Examination of the regional distribution of the inhaled iodine suggests that the deposition process critically initiates at a given point within conducting airways, including in distal conducting airways, and spreads to more peripheral regions from that point. This is illustrated in Fig. 5, where the regional deposition of iodine appears spatially correlated between 5 and 10 min images. This point is further illustrated in Fig. 9 where iodine deposition on conducting airway walls and at a branching point seems to increase with time (Fig. 9: *top*). A similar process may be occurring in distal sub-resolution airways in the lung periphery (Fig. 9: *bottom*). Turbulent mixing, and interception may have further contributed to deposition particularly in the proximal generations where flow velocity, hence the probability of turbulent flow is greater^14^. Together, these mechanisms may have caused the uneven deposition of aerosol in our study.

KES CT imaging has a few limitations. First, the dose of ionizing radiation is a limiting factor. However, recent studies have demonstrated that KES-CT can be performed with an air kerma of 40 mGy, which is within the range or only slightly above the range of clinically applicable doses^22^. Second, although the contrast sensitivity of KES CT is limited and inferior to radionuclide or fluorescent imaging, the smallest detectable concentration of iodine is 185 µg/ml, and even lower for gadolinium: 81 µg/ml^23^. The contrast sensitivity of KES CT is therefore sufficient for studying the regional lung deposition of aerosol particles. Also, due to the radiation beam geometry, the animal was maintained and imaged in the upright position, although aerosol administration can be performed in either supine, prone or upright postures. Finally, the observed iodine in the lung may originate from particles dissolved in the airway surface liquid and subsequently redistributed due to factors such as gravity and surface tension.

In conclusion, this study demonstrates for the first time the feasibility of synchrotron KES CT, for quantitative imaging of the regional lung deposition of aerosol particles, regional lung ventilation and morphology in preclinical models of both normal and diseased lungs, under controlled ventilation. Since these are all major factors determining regional lung aerosol deposition, we expect this imaging approach to bring new contributions to the understanding of lung aerosol delivery, targeting, and ultimately biological efficacy.

## Methods

### Animal preparation

The procedures for the animal care and the experiments were in accordance with the Directive 2010/63/EU of the European Parliament on the protection of animals used for scientific purposes and approved by the Internal Evaluation Committee for Animal Welfare in Research and the Safety Group of the European Synchrotron Radiation Facility (MD-429). The experiments were performed on 12 New Zealand White rabbits (2.9 ± 0.1 kg). Anesthesia was induced by IV injection of thiopental sodium (25 mg/kg) via a catheter (22 G) introduced into the marginal ear vein under local anesthesia (5% topical lidocaine). The animal was tracheostomised with a no. 3.5, Portex tube (Smiths medical, Kent, United Kingdom), and was mechanically ventilated using a custom system as described previously^24^. Briefly, the tracheal tube was connected to a T-piece supplied with constant adjustable gas flow. Solenoid valves controlled by the image acquisition electronic circuit allowed directing gas flow towards the animal during inspiration, or outwards upon passive expiration. Setting appropriate flows, inspiratory and expiratory durations allowed adjusting respiratory rate and minute ventilation. The animals were ventilated in with an average VT of 9 mL/kg; FIO2 of 0.21; I:E ratio of 1:2; a baseline PEEP of 0 cmH_2_O; and a respiratory rate set to obtain a PaCO_2_ of approximately 40 mmHg. The left carotid artery and jugular vein were catheterized for blood gas measurements and for IV methacholine administration, respectively. Anesthesia was then maintained with 0.1 mg/kg/h IV midazolam and analgesia was ensured by IV administration of fentanyl (50 µg/kg/h). After ensuring adequate anesthesia, continuous IV infusion of atracurium (1.0 mg/kg/h) was started. The animal was immobilized in the upright position in a custom-made plastic holder for imaging.

### Synchrotron radiation computed tomography imaging

The experiments were performed at the Biomedical Beamline of the European Synchrotron Radiation Facility (ESRF, Grenoble, France). The K-edge subtraction (KES) imaging technique allows quantitatively measuring the regional distribution of specific ventilation (s), while simultaneously visualizing lung morphology. A detailed description of the methodology and instrumental setup has been extensively described previously^24,25^. Briefly, this imaging technique uses 2 X-ray beams that can be tuned at slightly different energies above and below the K-edge, of a contrast element such as Xe (34.6 keV) or Iodine (33.2 keV), using a single cylindrically bent silicon (1 1 1) crystal in Laue geometry. X-rays from a synchrotron radiation source are required since, as opposed to standard X-ray sources, they allow the selection of monochromatic beams from the full X-ray spectrum while conserving enough intensity for imaging with sufficient temporal resolution. Two computed tomography images are thus simultaneously acquired during the inhalation of stable Xe gas or iodine-containing aerosol particles. The density due to the contrast element (Xe, I) can be separated from that of tissue, in each image voxel using a specifically developed computer algorithm explained in detail elsewhere^26^. The “Xe-density” or “I-density” images allow the direct quantitative measurement of these elements within the airways. A “tissue-density” image obtained from the same data allows the assessment of lung morphology.

### Measurement of Respiratory Mechanics

The airway and respiratory tissue parameters were assessed using the forced oscillation technique at low frequencies. These measurements were achieved by introducing a loudspeaker-generated small-amplitude (1 cmH_2_O peak to peak) pressure forcing signal (0.5–21 Hz) into the trachea via a polyethylene tube (100 cm length, 0.375 cm ID) while the mechanical ventilation was paused at end-expiration. The input impedance of the respiratory system (Zrs) was computed as described previously^27^. Three to five Zrs spectra were ensemble-averaged under each experimental condition. A model that includes airway resistance (Raw), inertances (Iaw) in series with constant-phase tissue compartments incorporating tissue damping (G) and elastance (H) was fitted to the averaged Zrs data^28^. Inertance, or the inertial component of impedance, reflects the masse of gas within the airways.

### Particle size measurement

The aerosol particle size was measured by laser diffraction using a particle size analyzer (Mastersizer X, Malvern Instruments, Malvern, UK). Eight independent measurements were performed in identical experimental conditions on the aerosolized iodine contrast-containing solution and the resulting median aerodynamic diameter (MAD) and geometric standard deviations were averaged.

### Study Protocol

The experiments were performed in 2 groups (n = 6 each); control and methacholine-induced bronchoconstriction (MCH). After a 10 min stabilization, a recruitment maneuver was performed (20 cmH_2_O, 10 s) followed by forced oscillatory measurements. Ten sequential synchrotron KES images were acquired, each preceded by 4 breaths of a (∼70% Xe in O_2_ gas mixture. These image sequences were then used to compute regional specific ventilation maps as described previously^24^. The Xe wash in images were obtained at 4 axial levels ranging from the 4^th^ to the 8^th^ intercostal space. The axial positions were standardized based on anatomic landmarks and the apex-diaphragm distance, measured on a thoracic projection image. The beam energies were then tuned to iodine K-edge by modifying the curvature of the monochromator crystal. In the control groups, a solution containing iodine contrast (Iomeprol 88 mg/ml in NaCl 0.9%) was aerosolized using an ultrasonic nebulizer (SAM LS2000, Villeneuve sur Lot, France). The aerosol was administered through the mechanical ventilation circuit for five minutes, after which 35 serial adjacent KES images were acquired with a slice thickness of 1 mm, spanning over 35 mm of lung height, ranging from the 4^th^ to the 8^th^ intercostal space. The aerosol was administered a second time for 5 minutes in all animals and Iodine-KES imaging was repeated. In some control animals, aerosol administration was repeated twice more in order to better visualize the kinetics of Iodine-bearing aerosol deposition in the lung. In the MCH group, iodine-containing aerosol was given following the same protocol, but after initiation of an IV infusion of MCH (10 µg/kg/min) which was maintained throughout imaging. At the end of the image acquisition, the animals were euthanized by IV injection of sodium thiopental (Dolethal 200 mg/ml, 5 ml; Vetoquinol, Lure, France).

### Image Analysis

Images were processed using the MATLAB programming package (Mathworks Inc., Natick, MA, USA) as described previously^9,24^. Lung tissue was selected within the tissue-density computed tomography images, by region-growing segmentation. The regional specific ventilation, defined as ventilation per gas volume within the voxel (s), was calculated from the time constant of the Xe wash-in using a single compartment model fit of Xe concentration vs. time^24^. A 5 × 5 pixel moving average window was applied to the Xe-density images prior to the model fit.

### Statistical Analysis

The scatters in the parameters were expressed by the standard deviation. The Shapiro-Wilk test was used to test data for normality. One-way repeated measures ANOVA was applied to evaluate the changes in respiratory mechanical and imaging parameters between the experimental conditions with control, and MCH as within-subject variables. Student’s t-test or the Mann-Whitney U test was used to compare physiological parameters between control and MCH groups, depending on normality of data distribution. The statistical analyses were conducted using SigmaPlot (version 13.0, Systat Software, Inc. Chicago, IL, USA). All statistical tests were carried out with a significance level of: p < 0.05.

### Availability of materials and data

The datasets analysed during the current study are available from the corresponding author on reasonable request.
